# Aggregation Propensity of the Human Proteome

**DOI:** 10.1371/journal.pcbi.1000199

**Published:** 2008-10-17

**Authors:** Elodie Monsellier, Matteo Ramazzotti, Niccolò Taddei, Fabrizio Chiti

**Affiliations:** 1 Dipartimento di Scienze Biochimiche, Università degli studi di Firenze, Florence, Italy; 2 Consorzio interuniversitrio “Istituto Nazionale Biostrutture e Biosistemi” (I.N.B.B.), Rome, Italy; National Cancer Institute, United States of America and Tel Aviv University, Israel

## Abstract

Formation of amyloid-like fibrils is involved in numerous human protein deposition diseases, but is also an intrinsic property of polypeptide chains in general. Progress achieved recently now allows the aggregation propensity of proteins to be analyzed over large scales. In this work we used a previously developed predictive algorithm to analyze the propensity of the 34,180 protein sequences of the human proteome to form amyloid-like fibrils. We show that long proteins have, on average, less intense aggregation peaks than short ones. Human proteins involved in protein deposition diseases do not differ extensively from the rest of the proteome, further demonstrating the generality of protein aggregation. We were also able to reproduce some of the results obtained with other algorithms, demonstrating that they do not depend on the type of computational tool employed. For example, proteins with different subcellular localizations were found to have different aggregation propensities, in relation to the various efficiencies of quality control mechanisms. Membrane proteins, intrinsically disordered proteins, and folded proteins were confirmed to have very different aggregation propensities, as a consequence of their different structures and cellular microenvironments. In addition, gatekeeper residues at strategic positions of the sequences were found to protect human proteins from aggregation. The results of these comparative analyses highlight the existence of intimate links between the propensity of proteins to form aggregates with β-structure and their biology. In particular, they emphasize the existence of a negative selection pressure that finely modulates protein sequences in order to adapt their aggregation propensity to their biological context.

## Author Summary

Amyloid-like fibrils are insoluble proteinaceous fibrillar aggregates with a characteristic structure (the cross-β core) that form and deposit in more than 40 pathological conditions in humans. These include Alzheimer's disease, Parkinson's disease, type II diabetes, and the spongiform encephalopathies. A number of proteins not involved in any disease can also form amyloid-like fibrils in vitro, suggesting that amyloid fibril formation is an intrinsic property of proteins in general. Recent efforts in understanding the physico-chemical grounds of amyloid fibril formation has led to the development of several algorithms, capable of predicting a number of aggregation-related parameters of a protein directly from its amino acid sequence. In order to study the predicted aggregation behavior of the human proteome, we have run one of these algorithms on the 34,180 human protein sequences. Our results demonstrate that molecular evolution has acted on protein sequences to finely modulate their aggregation propensities, depending on different parameters related to their in vivo environment. Together with cellular control mechanisms, this natural selection protects proteins from aggregation during their lifetime.

## Introduction

The conversion of peptides and proteins into insoluble fibrillar aggregates is the hallmark of *ca*. 40 human diseases [1]–[2]. It is now clear, however, that the formation of such well-organized fibrillar aggregates, generally referred to as amyloid fibrils when deposition occurs extracellularly, is not a characteristic of the few unfortunate sequences associated with protein deposition diseases, but a generic property of polypeptide chains [3]. This novel concept has raised the question as to how protein aggregation is prevented effectively in living organisms. Molecular chaperones and several dedicated cellular quality control mechanisms can fulfill this requirement [4]–[5]. However, it has also emerged that proteins have evolved numerous sequence and structural adaptations to counteract their natural tendency to aggregate into amyloid-like fibrils [6].

The generality of amyloid fibril formation has also suggested that this phenomenon may be governed by simple and rationalizable physicochemical factors, leading to the development of algorithms capable of predicting aggregation parameters of unstructured polypeptides directly from their amino acid sequence [7]–[14]. Computational methods based on atomistic description and/or molecular dynamics were also developed [15]–[20]. These algorithms have the potential to predict a number of aggregation-related parameters, including the aggregation rate or aggregation propensity of a polypeptide chain, the regions of the sequence that promote aggregation and the effect of mutations on the aggregation behavior.

The simplicity of the sequence-based algorithms allowed their application to the systematic analysis of all the protein sequences composing the proteomes of one or more living organisms [21]–[25]. By using this strategy, Serrano and co-workers demonstrated that intrinsically disordered proteins have a lower aggregation propensity than globular proteins [21]. The same group also showed that in proteins from *E. coli* positions flanking aggregation-promoting regions are enriched with residues with a low aggregation propensity, such as proline, arginine, lysine, glutamate and aspartate [22]. Interestingly, when the analysis is restricted to the most highly aggregation-promoting regions, only proline, lysine and arginine become dominant at these flanking positions [22]. The over-representation of such residues at these positions can result from their physicochemical properties, as well as from the ability of *E. coli* co-translational chaperones to recognize them when associated with hydrophobic stretches [22].

In another work the entire proteomes of *D. melanogaster*, *S. cerevisiae* and *C. elegans* were analyzed [23]. Proteins normally forming oligomeric complexes were found to have an aggregation score lower than those operating in a free form in all three organisms [23]. This was explained by considering that oligomer-forming proteins are at risk for aggregation as they constantly interact with other polypeptide chains. In addition, essential proteins were found to have a lower aggregation score than non-essential proteins, emphasizing the evolutionary pressure that has acted on the former to minimize their aggregation propensity [23]. Using another independently developed algorithm, Tartaglia and co-workers demonstrated that the average aggregation propensity of a proteome correlates inversely with the complexity and longevity of the related organism, underlining the importance of studying each organism independently [24]. The same authors found that in the proteome of the yeast *S. cerevisiae* proteins with different functions, as well as proteins featuring different subcellular localizations, have very different aggregation potentials [25].

In this work we use a previously developed algorithm [8],[11],[26] to analyze the 34,180 protein sequences of the human proteome. The algorithm is based on simple characteristics of the primary sequence, such as hydrophobicity, β-sheet propensity and charge, previously recognized to be important determinants of the aggregation process [7]. It predicts the fibril elongation rate of an initially unstructured polypeptide chain as well as the regions of the sequence that promote its aggregation, and has been extensively validated against experimental data [8],[11],[26]. This algorithm represents therefore a valid and straightforward computational tool to quantify the intrinsic aggregation propensities of a large quantity of protein sequences and identify the aggregation-promoting regions within them.

The application of this computational tool to the human proteome enabled us to recognize unprecedented features, including an inverse correlation between aggregation propensity and protein length, and a discrepancy between the aggregation propensities of proteins taking the secretory pathway (operating in the endoplasmic reticulum, Golgi apparatus, lysosomes and extracellular media) and those operating in other intracellular compartments (nucleus, mitochondria, ribosomes, cytoskeleton). We were also able to reproduce some of the results obtained with other algorithms, demonstrating that the results obtained do not depend on the type of computational tool employed, and cross-validating the different existing algorithms. In addition, the previously published results have in general been obtained by studying prokaryotes like *E. coli* or low-complexity eukaryotes like *S. cerevisiae*, whereas our analysis focuses entirely on the human proteome. Our results also show that different structural subpopulations of the human proteome have actually different average aggregation propensities, whereas proteins involved in protein deposition diseases do not differ extensively from the human proteome taken at a whole in terms of aggregation propensity. Taken together, these results lend further support to the view that modulation of the aggregation propensity has been a driving force in protein evolution. It also helps identify the categories of human proteins that are at risk for aggregation and need a more strict control by the cellular machinery.

## Results/Discussion

### Determination of the Aggregation Propensity of All Proteins from the Human Proteome

**Figure 1:**
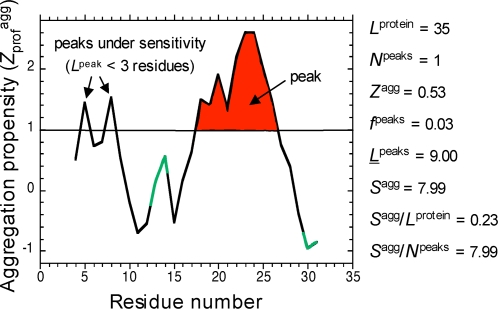
Definition of the aggregation parameters calculated for a sequence. The aggregation propensity profile and the parameters shown in the figure refer to the peptide A-Dan, used here as an example. Red area: surface of the aggregation peaks (*S* ^agg^); green: flanking positions.

We have used a predictive algorithm [11],[26] to calculate the aggregation propensity of every protein sequence of the human proteome. A set of parameters were calculated for each of the 34,180 sequences (Figure 1; see also Methods, Protocol S1, and Figure S2):

- the average aggregation propensity of the sequence (*Z* ^agg^) and the intrinsic aggregation propensity profile (*Z*_prof_^agg^), i.e. the variation of aggregation propensity across the sequence;
- the frequency of the aggregation peaks (*f* ^peaks^), i.e. the number of stretches of at least 3 consecutive residues with an aggregation propensity *Z*_i_^agg^ higher than 1, divided by the number of residues in the sequence;
- the average length of all the aggregation peaks present in the sequence (*L* ^peaks^);
- the area of each aggregation peak, i.e. the surface under the peak that lies above the threshold of *Z*_i_^agg^ = 1 (*S* ^agg^); *S* ^agg^ was then normalized by both the protein length (*S* ^agg^/*L* ^protein^) and the number of peaks (*S* ^agg^/*N* ^peaks^).

All membrane intrinsic proteins (5,279 sequences) were removed from the database and analyzed separately, as they differ significantly from the non-membrane proteins in terms of charge and hydrophobicity [27], parameters that are preponderant in determining the aggregation propensity of a protein [7]. The distributions of the aggregation parameters over the human proteome cleared from the membrane protein were analyzed (see Figure S1). The protein length (*L* ^protein^) and the aggregation propensity (*Z* ^agg^) have a log-normal and a normal distribution, respectively. The *f* ^peaks^, *L* ^peaks^, *S* ^agg^/*L* ^protein^ and *S* ^agg^/*N* ^peaks^ parameters do not have well-defined distributions, but approximate to log-normal distributions when proteins devoid of aggregation peaks are not taken into account.

### Longer Proteins Have Less Pronounced Aggregation Peaks

**Figure 2:**
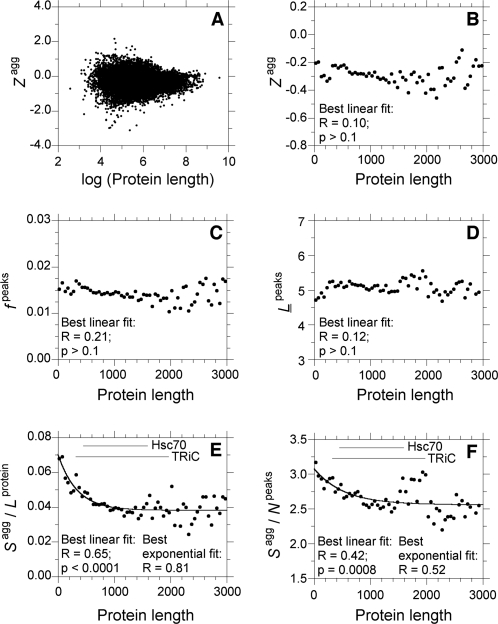
Dependence of the aggregation parameters on protein length. (A) All proteins are reported with their individual values of *Z* ^agg^ and protein length. For graphical convenience, the log of the protein length is represented. (B–F) Each point represents the average value over all the sequences having a length comprised in an interval of 50 residues. The membrane proteins are excluded from all the analyses reported in the figure. Solid lines (E–F) represent the best fits to an exponential function. The sizes of the substrates typically targeted by the chaperones Hsc70 and TriC [34]–[35] are indicated as horizontal solid lines (E–F).

We first looked at the dependence of the aggregation parameters on protein length (Figure 2). When the aggregation propensities *Z* ^agg^ of all the non-membrane proteins of the human proteome are plotted against their lengths in amino acid residues, no significant correlation is apparent (Figure 2A). To limit the influence of the outlying longest proteins on the χ^2^ calculation of the linear correlation, polypeptide chains longer than 3,000 residues (0.6% of the proteome) were excluded from the analysis. In addition, for each protein length interval of 50 residues, a single *Z* ^agg^ value was determined as the average value of all proteins falling in that interval. This allows the different intervals to have similar weights in the correlation, regardless of the number of proteins present in each case. Following these procedures, no correlation is found between *Z* ^agg^ and protein length (Figure 2B). The absence of correlation persists when different interval lengths or length thresholds are used for the analysis. Similarly to *Z* ^agg^, the frequency of aggregation peaks (*f* ^peaks^) and their average length (*L* ^peaks^) do not change with protein length (Figure 2C and 2D). On the contrary, the total surface of the aggregation peaks normalized either by the protein length (*S* ^agg^/*L* ^protein^) or by the number of peaks (*S* ^agg^/*N* ^peaks^), correlate inversely with protein length (Figure 2E and 2F). The *R* and *p* values obtained by analyzing the plots with a best-fitting procedure and a linear function indicate that such correlations are significant (Figure 2E and 2F). However, the dependence of *S* ^agg^/*L* ^protein^ and *S* ^agg^/*N* ^peaks^ on protein length seems to be rather exponential or hyperbolic (Figure 2E and 2F). Again, these results appear to be robust and independent of the calculation method. From this analysis it can be concluded that long proteins have, on average, less effective aggregation-promoting regions than those present in short ones.

These results imply that while aggregation-prone residues are present to similar extents in long and short proteins, their clustering within short segments of the sequence is less pronounced in long proteins. This feature has been demonstrated to be an important determinant of the aggregation rate of a model unstructured polypeptide chain [26], as well as of a set of proteins (G-G. Tartaglia and M. Vendruscolo, personal communication). For example, in apoMb_1–29_, a model unstructured polypeptide that encompasses the first 29 residues of horse heart apomyoglobin, the clustering of aggregation-prone residues in a narrow region of the sequence was found to enhance dramatically the aggregation rate [26]. Why have long proteins evolved to have less pronounced aggregation peaks and, as a consequence, slower aggregation rates? Reduced aggregation peaks could counteract the fact that long proteins have, with respect to short ones, pI closer to neutrality [28], a higher number of stretches of alternating hydrophobic-hydrophilic residues [29], and slower folding rates [30]. All these features have indeed been demonstrated to increase the aggregation propensity of an unstructured polypeptide [7], [31]–[33].

Interestingly, it has been shown that two major cytosolic mammalian chaperones, namely Hsc70 and the chaperonin TriC, interact preferentially with large proteins [34]. A large fraction of the Hsc70 protein substrates is heavier than 50 kDa [34]. The chaperonin TRiC interacts predominantly with proteins between 30 and 60 kDa, but also with several larger proteins, with the 2000-residue myosin heavy chain representing its heavier identified substrate [34]–[35]. It is noteworthy that the lower size limits of Hsc70 and TriC substrates correspond approximately to the inflection points of the exponential dependences of the parameters *S* ^agg^/*L* ^protein^ and *S* ^agg^/*N* ^peaks^ on protein length (Figure 2E and 2F). In eukaryotes long proteins also tend to be expressed at lower levels than short ones [36]–[37], which reduces their local concentration and thus their susceptibility to aggregate.

This comparative analysis suggests that diverse complementary mechanisms could have been developed through evolution to counteract the particular susceptibility of long proteins to aggregate. In addition to an assisted folding by chaperones and reduced expression levels, our results show that long protein sequences themselves have probably been constrained by evolution to reduce their intrinsic aggregation propensity, through an attenuation of their aggregation-prone regions.

### Proteins with Different Subcellular Localization Have Different Aggregation Propensities

**Figure 3:**
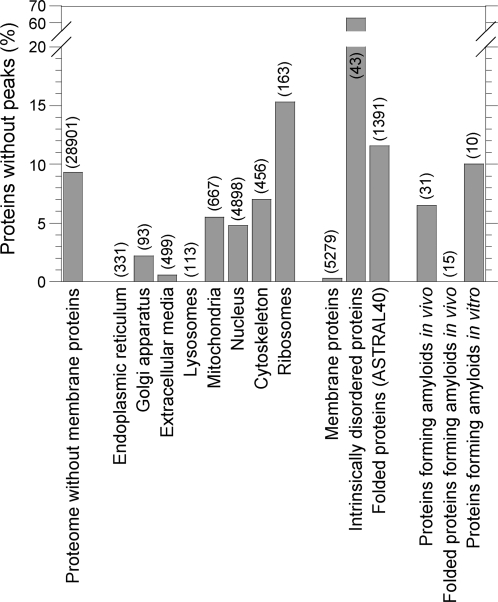
Percentages of proteins without aggregation peaks in different populations. The number of sequences composing each population is given in parentheses.

**Figure 4:**
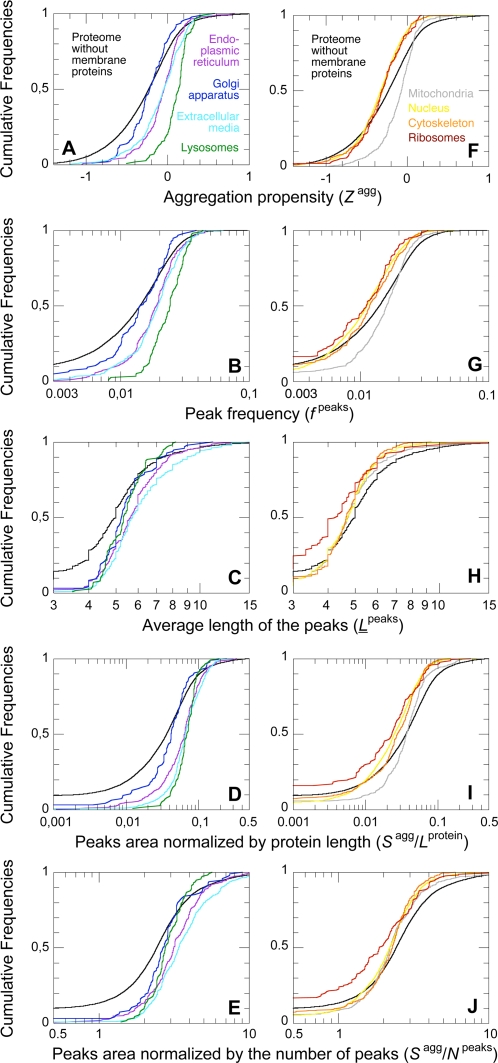
Cumulative distributions of the aggregation parameters in populations regrouping proteins from different subcellular localizations. Black: proteome without membrane proteins (28,901 sequences); purple: proteins from the endoplasmic reticulum (331 sequences); dark blue: proteins from the Golgi apparatus (93 sequences); light blue: proteins from the extracellular media (499 sequences); green: proteins from lysosomes (113 sequences); grey: mitochondrial proteins (667 sequences); yellow: nuclear proteins (4,898 sequences); orange: proteins of the cytoskeleton (456 sequences); red: ribosomal proteins (163 sequences). The membrane proteins were excluded from all the subcellular populations analyzed.

We then compared the distributions of the various aggregation parameters in proteins from different subcellular localizations (Figures 3 and 4; Table 1, lines 1–8). Membrane proteins were excluded from the analysis. Subcellular localizations can be divided in two groups. The proteins that take the secretion pathway are more prone to aggregate than the human non-membrane proteins taken as a whole (Figure 3; Figure 4A–4E; Table 1, lines 1–4). Indeed, all the aggregation parameters of proteins from the endoplasmic reticulum, the extracellular media and the lysosomes are systematically higher than the ones of the human non-membrane proteins in general (Figure 4A–4E; Table 1, lines 1,3,4). None of the proteins from the endoplasmic reticulum and the lysosomes and only 0.6% of the proteins from the extracellular media are devoid of aggregation peaks (Figure 3). Golgi proteins are also more prone to aggregate than human non-membrane proteins (Figure 3; Figure 4A–4E; Table 1, line 2). In this case the discrepancy is less marked, probably due to the lower amount of sequences analyzed. On the contrary, intracellular districts like the nucleus, cytoskeleton and ribosomes contain proteins with particularly low propensities to aggregate, according to every parameter analyzed (Figure 3; Figure 4F–4J; Table 1, lines 6–8). The case of the mitochondria is intermediate between these two cases (Figure 4F–4J; Table 1, line 5). The peculiarity of this organelle in terms of aggregation propensity may reflect its prokaryotic origin. To exclude a possible bias due to the presence of specific signal peptides in the protein sequences of some compartments, the analysis was repeated after removing signal peptides from the protein sequences contained in our databases (see Methods). This correction did not modify the above-mentioned results (Table 1, lines 1–8).

**Table 1:** Comparisons between the aggregation parameters in different populations.

Analyzed population	Reference population	*L* ^protein^	*Z* ^agg^	*f* ^peaks^	*L* ^peaks^	*S* ^agg^/*L* ^protein^	*S* ^agg^/*N* ^peaks^
Endoplasmic reticulum^a^	all except membrane proteins	+++	+++	+++	+++	+++	+++
Golgi apparatus^a^	all except membrane proteins	+++	n.s.	n.s.	+++	n.s.^c^	++
Extracellular media^a^	all except membrane proteins	− − −	+++	+++	+++	+++	+++
Lysosomes^a^	all except membrane proteins	+++	+++	+++	+++	+++	+++
Mitochondria^a^	all except membrane proteins	− − −	+++	+++	− −	n.s.^c^	− −
Nucleus^a^	all except membrane proteins	+++	− − −	− − −	− − −	− − −	− − −
Cytoskeleton^a^	all except membrane proteins	+++	− − −	− − −	− − −	− − −	− − −
Ribosomes^a^	all except membrane proteins	− − −	− − −	− − −	− − −	− − −	− − −
Membrane proteins	all	+++	+++	+++	+++	+++	+++
Intrinsically disordered proteins	all except membrane proteins	− − −	− − −	− − −	− − −	− − −	− − −
Folded proteins	all except membrane proteins	− − −	+++	+++	− − −	+++	− − −
Proteins forming fibrillar aggregates in vivo^b^	all except membrane proteins	−	+++	++	n.s.	+^d^	n.s.
Proteins from the extracellular media forming fibrillar aggregates in vivo^b^	extracellular media	− − −	+^d^	n.s.	n.s.	n.s.	− −
Proteins from the cytoskeleton forming fibrillar aggregates in vivo^b^	cytoskeleton	n.s.	n.s.	n.s.	n.s.	n.s.	−d
Folded proteins forming fibrillar aggregates in vivo^b^	folded proteins	n.s.	n.s.	n.s.	n.s.	n.s.	n.s.
Folded proteins forming fibrillar aggregates in vitro	folded proteins	−	n.s.	n.s.	n.s.	n.s.	n.s.

The distributions of the aggregation parameter values of the analyzed population are compared to the ones of the reference population using statistical tests (see Methods).

+++ and −−− indicate that the analyzed population has a distribution significantly (p<0.001) shifted to higher or lower values than the reference population in the statistical tests performed, respectively. ++ and −−, idem (p<0.01). + and −, idem (p<0.05).

n.s., the distributions of the analyzed and reference populations are not significantly different (p>0.05).

a The results remain unchanged when the sequences without signal peptides of the corresponding subcellular districts (membrane proteins excluded) are compared with a reference database composed of all the human non-membrane protein sequences without the identified signal peptides.

b Proteins forming amyloid fibrils and intracellular inclusions with amyloid-like characteristics.

c The distributions of the two populations differ significantly although their median values are not significantly different (significant differences in the Kolmogorov-Smirnov test and not in the Mann-Whitney test).

d The difference was significant in the Mann-Whitney test but not in the Kolmogorov-Smirnov test (parameter lacking a defined distribution).

How can the discrepancy between proteins taking the secretion pathway and proteins confined to the intracellular media be explained? Since proteins targeted for secretion or for other cellular compartments on the way to secretion operate in areas where chaperones are poorly represented, several strict quality control mechanisms check whether such proteins are properly folded or not [5],[38]. This high level of extrinsic cellular control balances and enables proteins with high intrinsic aggregation propensities. On the contrary, in *E. coli*, periplasmic proteins have been demonstrated to be more resistant to aggregation than cytoplasmic ones, in relation to the paucity of quality control machineries and molecular chaperones in the bacterial periplasm [39]. The differences observed in the aggregation propensities of proteins from different compartments can also partially reflect the different proportions of intrinsically disordered proteins in these compartments (see below; [40]), or differences in the conditions in which proteins fold. Different organelles can differ in terms of pH, redox potential, presence of proteases, molecular crowding, and types and abundance of chaperones [41]–[42]. In particular, it has been shown experimentally that the nuclear compartment creates an environment that renders proteins more prone to denaturate [42]–[43], which is in good agreement with our data. Thus, protein sequences would be precisely adapted to the conditions in which they evolve in vivo.

Remarkably, using a different predictive algorithm applied on the proteome of the yeast *S. cerevisiae*, Tartaglia and colleagues ordered the subcellular areas according to the β-aggregation propensity of their proteins and obtained the same ranking [25]. The agreement between these two analyses on evolutionary distant eukaryotic organisms, such as *S. cerevisiae* and *H. sapiens*, emphasizes the generality of the observed phenomena. Evolution seems to have modulated the average aggregation propensities of different subcellular areas in a similar manner in such distantly related organisms.

### Membrane Proteins, Intrinsically Disordered Proteins, and Folded Proteins Have Different Aggregation Propensities

**Figure 5:**
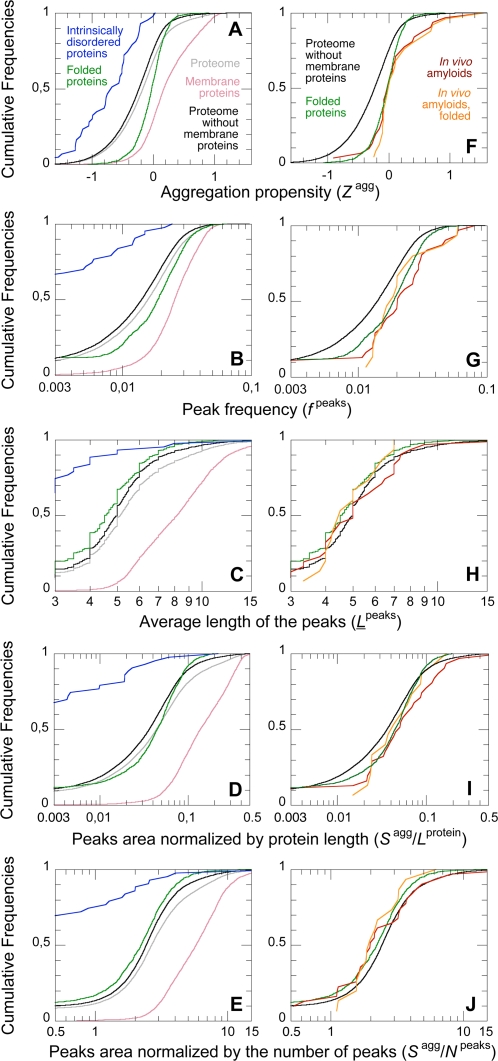
Cumulative distributions of the aggregation parameters in different populations. Grey: all proteome (34,180 sequences); pink: membrane proteins (5,279 sequences); black: proteome without membrane proteins (28,901 sequences); green: folded proteins (ASTRAL40 database; 1,391 sequences); blue: intrinsically disordered proteins (43 sequences); red: proteins forming amyloids or related intracellular inclusions in vivo and associated with human diseases (31 sequences); orange: folded proteins forming amyloids or related intracellular inclusions in vivo and associated with human diseases (15 sequences).

We performed the same comparative analysis on different populations of the human proteome. Membrane proteins have significantly higher values of every aggregation parameter analyzed than the whole human proteome (Figure 5A–5E; Table 1, line 9). Moreover, only 0.3% of membrane proteins do not contain aggregation peaks, whereas this fraction rises to 7.9% for the whole human proteome (Figure 3). Thus, membrane proteins constitute a very distinct and peculiar group.

The intrinsically disordered proteins form another distinct group. In our analysis we considered proteins or protein segments (>40 residues) that were experimentally shown to be intrinsically disordered (43 sequences; see Methods for details). For all the parameters studied, the values of the intrinsically disordered proteins were particularly low, and significantly lower than the corresponding parameters from the reference database (Figure 5A–5E; Table 1, line 10). A majority of them (63%) do not contain any aggregation peak at all (Figure 3).

We then used the SCOP-derived database ASTRAL40 [44] to have an experimentally determined population of folded human proteins (1,391 sequences). Proteins from the ASTRAL40 database have higher values of *Z* ^agg^, *f* ^peaks^ and *S* ^agg^/*L* ^protein^ than the reference database, most probably due to the significant proportion of intrinsically disordered proteins in the latter (Figure 5A, 5B, and 5D; Table 1, line 11). However, such folded proteins have all aggregation parameters significantly higher than intrinsically disordered proteins (Figure 5A–5E). Linding and co-workers also compared intrinsically folded and disordered proteins [21]. The analysis was performed with a different algorithm for the prediction of β-aggregation, using only experimentally characterized proteins and with no restriction on the organism of origin [21]. Following this approach these authors obtained a similar separation between intrinsically folded and disordered proteins, and similar values for the frequency of aggregation-prone regions and percentage of proteins without aggregation-prone regions in these two populations [21].

The differences in the aggregation propensities between the membrane, folded and intrinsically disordered proteins could possibly arise from their different structures. In membrane proteins, pronounced aggregation peaks are perfectly soluble in the lipid bilayer, as they are mostly hydrophobic [45]. Folded proteins can also tolerate aggregation-prone regions, because such segments are generally buried in the hydrophobic core of the protein, and thus protected from inter-molecular interactions [6],[21],[46]. On the contrary, intrinsically disordered polypeptides expose the whole backbone to the solvent, and thus need peculiar sequence adaptations to reduce their aggregation propensities [6],[47].

### Fibril Forming Proteins Do Not Differ Extensively from the Other Human Proteins

Proteins that were found to form amyloid fibrils or structurally related intracellular inclusions in the context of human protein deposition diseases were also analyzed (31 sequences; see Protocol S1 for a list). Proteins containing a poly-Gln stretch like huntingtin were excluded from this list, as they aggregate through a different mechanism [48]. Disease-related proteins are not found to be systematically different from the other human non-membrane proteins. Their *Z* ^agg^ and *f* ^peaks^ values are significantly higher than those of the reference database (Figure 5F–5G; Table 1, line 12). However, the distributions of *L* ^peaks^ and *S* ^agg^/*N* ^peaks^ are not significantly different (Figure 5H and 5J; Table 1, line 12). The distributions of *S* ^agg^/*L* ^protein^ differ to some extent, but we cannot exclude that such difference arises from the shorter average length of disease-related proteins with respect to the reference proteins (Figure 5I; Table 1, line 12).

As described above proteins, from different subcellular compartments have different aggregation propensities. We thus compared groups of proteins forming fibrillar aggregates in vivo with proteins from the corresponding subcellular districts. This was performed on fibril-forming proteins from the extracellular media (23 sequences; Table 1, line 13) and from the cytoskeleton (3 sequences; Table 1, line 14), comparing them with all extracellular and cytoskeleton proteins, respectively. In these two comparative analyses the differences between proteins forming fibrils in vivo and corresponding human proteins are not significant (Table 1, lines 13–14). Similar comparative analyses could not be carried out for the other subcellular compartments as the numbers of fibril-forming sequences were insufficient for statistical analyses.

Finally, we compared the folded proteins associated with diseases (15 sequences; see Protocol S1 for a list) with the reference database of folded human proteins (ASTRAL40). None of the aggregation parameters appeared to be significantly different (Figure 5F–5I; Table 1, line 15). Similarly, 10 non-disease folded proteins shown to form amyloid-like fibrils in vitro (see Protocol S1 for a list) do not differ for any of the aggregation parameters from the folded proteins of the ASTRAL40 database (Table 1, line 16).

All these analyses support the view that no fundamental differences exist in terms of intrinsic aggregation propensity between proteins related to protein deposition diseases and the remainder of the human proteome. This result cannot be due to an inability of the used algorithm to detect differences in the aggregation propensities of two populations of proteins when they actually exist, as demonstrated by the remarkable discrepancies observed between proteins from different subcellular compartments (Figure 4) and between membrane, folded and intrinsically disordered proteins (Figure 5). On the contrary, this finding explains why proteins that are not associated with recognized protein deposition diseases also have an inherent ability to form amyloid-like fibrils in vitro [3],[49]. It also suggests that the reason why only a limited number of human proteins give rise to protein deposition diseases has to be sought in the biology of such proteins, rather than in specific traits dictated by their amino acid sequences. These concepts have been proposed for the first time almost a decade ago following individual experimental observations [3]. The result obtained here at the genomic scale is a strong argument to support them.

### Human Proteins Have Evolved To Secure Gatekeeper Residues at Positions Flanking the Peaks

**Figure 6:**
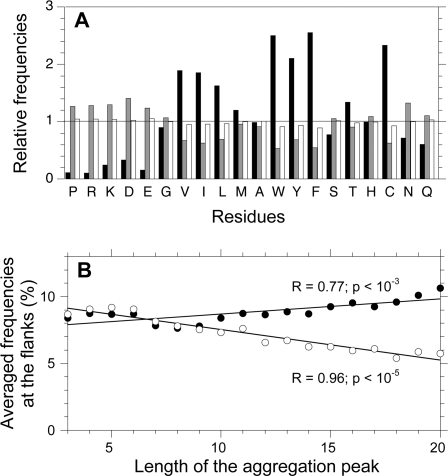
Gatekeeper residues in the human proteome. (A) Amino acid frequencies at different positions, relative to their global frequencies in the human proteome. A relative frequency of 1.0 for a given residue at a given position means that the residue occupies that position with a frequency identical to that in the whole human proteome. Black: inside the aggregation peaks; grey: at the flanking positions; white: outside the aggregation peaks and far from the flanks (“valleys”). (B) Dependence of the frequencies of the gatekeepers at the flanks on the length of the aggregation peak. Filled circles: average frequencies of Pro, Arg and Lys; empty circles: average frequencies of Asp and Glu. The membrane proteins are removed from the database.

We analyzed the amino acid composition in the aggregation peaks, their flanks, i.e. the regions of the sequence immediately preceding and following the peaks (see Methods for an accurate definition of the flanks) and the rest of the sequence (“valleys”). As expected, residues with high intrinsic aggregation propensity, like Trp, Phe, Tyr, Cys, Val, Ile and Leu, are mostly found inside the peaks, whereas residues with low aggregation propensity, like Pro, Arg, Lys, Asp and Glu, are poorly represented in the peaks (Figure 6A). The latter are particularly highly represented at the flanks, where they are more frequent than in both the peaks and valleys (Figure 6A). On the contrary, residues with high intrinsic aggregation propensity are poorly represented at the flanks and even less frequent than in the valleys (Figure 6A). Thus, our definition of flanks corresponds to a portion of the protein sequences with a very specific amino acid residue composition.

Strikingly, the relative distributions of Pro, Arg, Lys, Asp and Glu at the flanking positions change with peak length. While Pro, Arg and Lys frequencies at the flanks increase significantly with peak length, Asp and Glu frequencies decrease (Figure 6B). Rousseau and colleagues obtained similar results on the *E. coli* proteome, using TANGO as a predictive algorithm of β-aggregation propensity [22]. The full agreement between the two algorithms, even after a fine analysis such as the frequency of specific residues at flanking positions and their dependence on peak length, offers a cross-validation of both computational methods and underlines the generality of the observation.

Thus, our results confirm the role of Pro, Arg and Lys as “gatekeeper residues” at the flanks of the aggregation peaks, protecting proteins with particularly extended peaks from aggregation. This specific role of these residues can be rationalized. Pro is conformationally constrained and thus an efficient breaker of β-structures. Arg and Lys are charged, and their long and flexible side-chains make the aggregation process entropically disadvantageous. Moreover, Arg and Lys have been shown to be specifically recognized by the most common chaperones when associated with hydrophobic stretches (reviewed in [22]).

### Conclusions

The analysis of the human proteome presented here has revealed that protein sequences have been constrained by evolution to finely modulate their aggregation propensity depending on their length, subcellular localization, and conformation. It has revealed a striking synergy between the evolution of protein sequences and biology. Modulation of the intrinsic aggregation propensity of protein sequences by molecular evolution on the one hand, and cellular protective mechanisms on the other hand, act as complementary strategies to prevent proteins to aggregate during their lifetime. The opportunities offered by a bioinformatics approach that uses experimentally tested and cross-validated algorithms are enormous. They will offer in the future new strategies not just to understand the link between protein aggregation and evolution, but also to learn the “tricks” set up by Nature to effectively control protein aggregation in highly crowded environments of living organisms.

## Methods

### Datasets

All the datasets cited in this work are available as Protocol S1 or upon request. The “entire human proteins” dataset has been downloaded from the ftp site of the National Center for Biotechnology Information (NCBI) as fasta-formatted translations of an mRNA collection (*RefSeq* database). The “intrinsically disordered proteins” dataset has been extracted as fasta-formatted sequences from the Database of Protein Disorder (DisProt), release 3.5 [50] using “homo sapiens” as a keyword for the “search by source organism” filter. Swiss-Prot based accession numbers were synchronized with the “entire human proteins” dataset (with NCBI based protein identifiers, GIs) with automated local BLAST searches [51]. The dataset was then adjusted to include both fully disordered proteins and non-redundant non-overlapping protein fragments of length >40 residues extracted from full sequences of partially disordered proteins. The “verified structured proteins” dataset has been obtained as fasta-formatted sequences from the ASTRAL40 database, release 1.71 [44]. Each protein in this dataset shares less than 40% sequence identity with all other proteins.

Information on protein subcellular localization has been extracted from Gene Ontology (GO) annotations [52]. Subgroups were constructed according to the following GO terms: integral to membrane (GO∶0016021; 5279 sequences), lysosomes (GO∶0005764; 113 sequences), nucleus (GO∶0005634; 4898 sequences), mitochondria (GO∶0005739; 667 sequences), Golgi apparatus (GO∶0005794; 93 sequences), endoplasmic reticulum (GO∶0005783; 331 sequences), cytoskeleton (GO∶0005856; 456 sequences), ribosomes (GO∶0005840; 163 sequences) and extracellular media (GO∶0005615; 499 sequences).

In a second time, all the above-mentioned datasets were rebuilt by removing signal peptides as a possible source of bias due to their peculiar amino acid composition. The position and extension of the signal peptides were taken, when available, from NCBI Protein database by an automatic and systematic analysis of protein annotations.

Other datasets include: proteins forming amyloid fibrils or intracellular inclusions with amyloid-like characteristics, all related to protein deposition diseases (with the exception of proteins containing a poly-Gln segment as they aggregate through a different mechanism); a subpopulation of these proteins containing only the folded ones; proteins forming amyloid-like fibrils in vitro, but unrelated to protein deposition diseases. These databases have been constructed by extensive search in literature and are listed in Protocol S1.

### Determination of Aggregation Parameters

The parameters *Z* ^agg^, *Z*_prof_^agg^, *f* ^peaks^, *L* ^peaks^ and *S* ^agg^ have been determined for each protein sequence as described in Protocol S1, and previously [11],[26].

### Flanking Regions and Gatekeepers

The definition of flanking regions has been accurately refined by a recursive procedure aimed at avoiding overlaps of flanks with neighbor peaks. The algorithm we used has two parameters for defining both the position (*F* ^p^) and length (*F* ^l^) of the flank. Given a region of the sequence defined as the pattern oooooôˆˆˆˆoooooo, (ˆ = residue with *z*_i_^agg^≥1, o = residue with *z*_i_^agg^<1), *F* ^p^ defines the starting position of the flank from both extremities of the peak and *F* ^l^ defines the length of the flank at both extremities. The optimized values were found to be *F* ^p^ = −3 (the flanks start from the residue o that is 3 residues distant from the first, or last, residueˆ) and *F* ^l^ = 3 (see Figure 1, or consider the pattern oooooôˆˆˆˆoooooo, where flanks are underlined). These values were valid for peaks of every length.

Amino acid compositions of peaks, flanks, peaks-free regions and whole sequence were calculated, for each naturally occurring amino acid, according to:
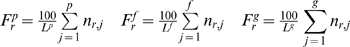
(1)where *F*_r_^p^, *F*_r_^f^, *F*_r_^g^ are the percentages of the *r* ^th^ amino acid residue in peaks, flanks and global respectively; *p*, *f* and *g* are the number of peaks, flanks and protein, respectively; and *L^p^* ,*L^f^* and *L^g^* are the total peak, flank and protein length calculated over the whole dataset, respectively.

### Statistics

The distributions of the aggregation parameters of two different protein populations were compared using the Student, Mann-Whitney and Kolmogorov-Smirnov tests for *Z* ^agg^ and the log of *L* ^protein^ (normal distributions), and Mann-Whitney and Kolmogorov-Smirnov tests for the other parameters studied (*f* ^peaks^, *L* ^peaks^, *S* ^agg^/*L* ^protein^ and *S* ^agg^/*N* ^peaks^).

Some of the parameters studied have a log-normal distribution, and have to be statistically analyzed in a different way than parameters with a normal distribution. For a normally distributed parameter X, the interval μ(X)±SD(X) (where μ(X) and SD(X) are the arithmetic mean and associated standard deviation of X, respectively) covers a probability of 68.3%. For a parameter Y having a log-normal distribution, the same probability is covered by μ[log(Y)]±SD[log(Y)] or by μ*(Y) •/SD*(Y), where μ*(Y) = e^μ[log(Y)]^ is the geometric mean of Y, SD*(Y) = e^SD[log(Y)]^ is the multiplicative standard deviation of Y, and •/means “times/divided by”. The importance of log-normal distributions in biology and the way to analyze them were previously discussed [53].

## Supporting Information

Figure S1 Distributions of the aggregation parameters in the human proteome. All the non-membrane proteins are considered in the analysis (28,901 sequences). For parameters that do not have a normal distribution, their log-normal distribution is given in insets.
(1.43 MB TIF)

Figure S2 Independence of μ_agg_ (A) and σ_agg_ (B) on protein length.
(0.22 MB TIF)

Protocol S1 Supporting Information
(0.12 MB RTF)
