# Supplementary figures and images for: Aggregation Propensity of the Human Proteome
Source: PLoS Comput Biol. 2008 Oct 17;4(10):e1000199. doi: 10.1371/journal.pcbi.1000199 (PMC2557143; doi:10.1371/journal.pcbi.1000199)

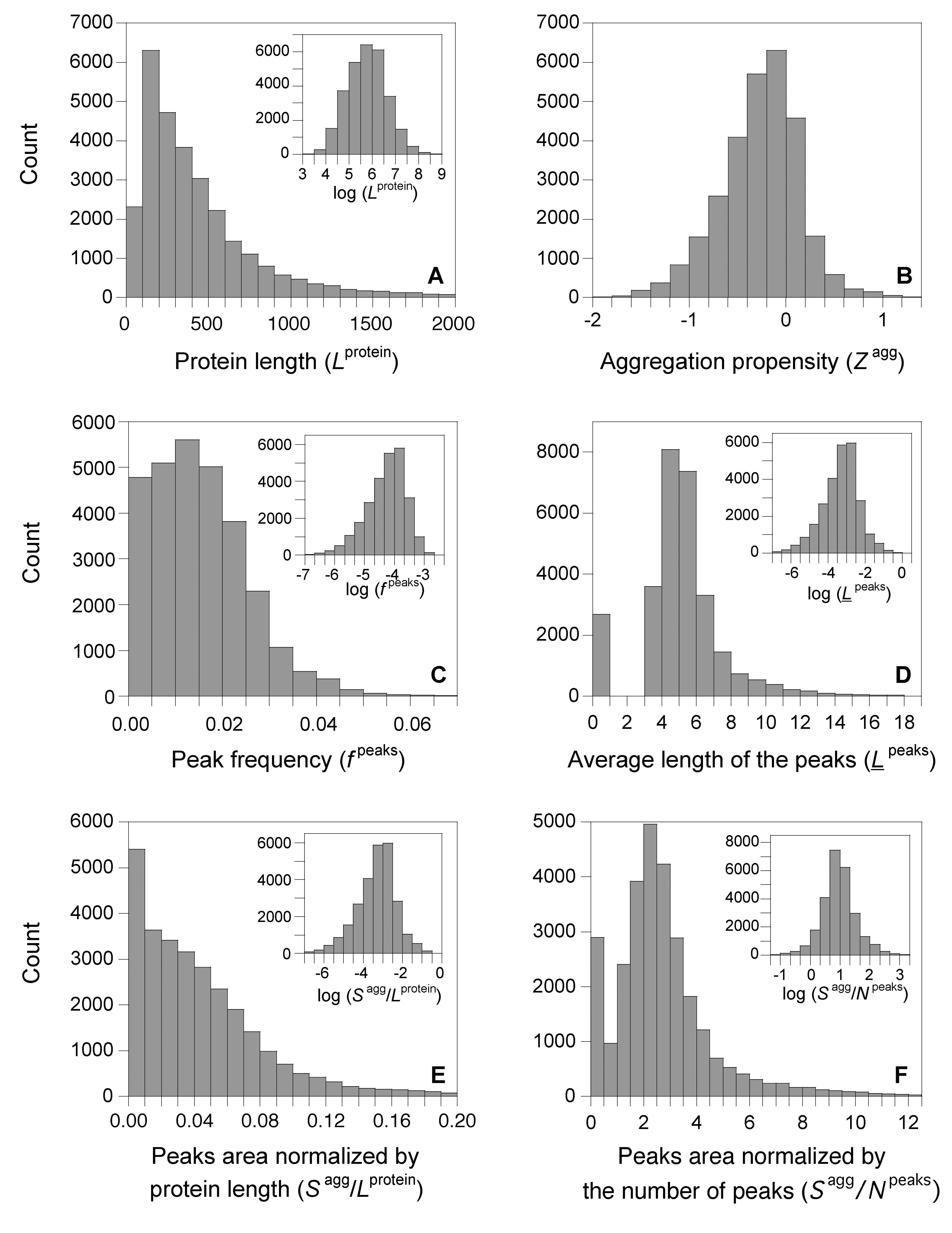

Supplement: Figure S1 — Distributions of the aggregation parameters in the human proteome. All the non-membrane proteins are considered in the analysis (28,901 sequences). For parameters that do not have a normal distribution, their log-normal distribution is given in insets. (1.43 MB TIF) [file pcbi.1000199.s001.tif]

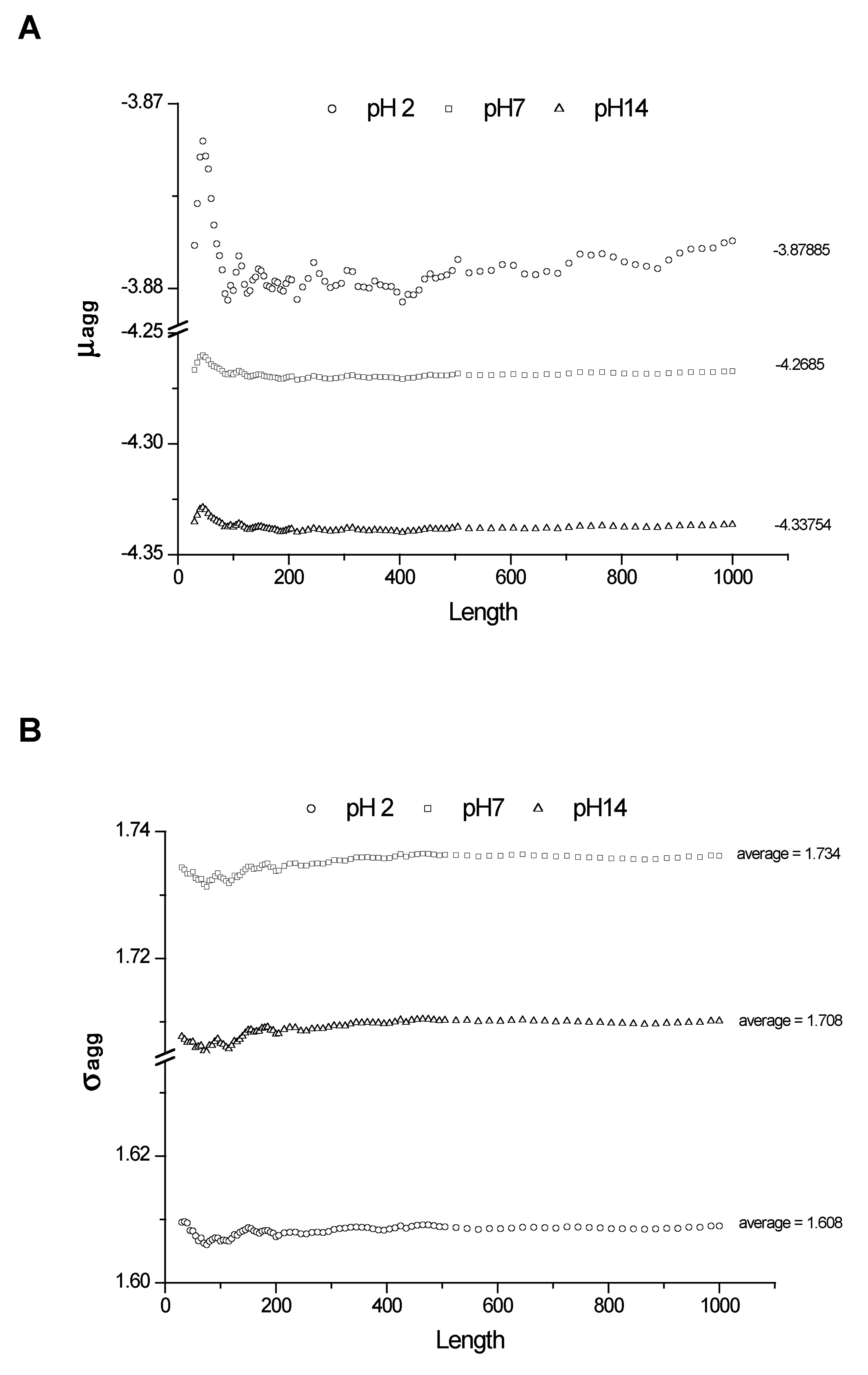

Supplement: Figure S2 — Independence of μagg (A) and σagg (B) on protein length. (0.22 MB TIF) [file pcbi.1000199.s002.tif]
