# Supplementary material for: Aggregation Propensity of the Human Proteome
Source: PLoS Comput Biol. 2008 Oct 17;4(10):e1000199. doi: 10.1371/journal.pcbi.1000199 (PMC2557143; doi:10.1371/journal.pcbi.1000199)
Supplement: Protocol S1 — Supporting Information (0.12 MB RTF) [file pcbi.1000199.s003.rtf]

Protocol S1

Determination of the intrinsic aggregation propensity (pagg, Pagg, Zagg)
The aggregation propensity of each human protein has been calculated following the previously described algorithm [1,2]. The intrinsic aggregation propensities (pagg) at pH 7.0 of each of the 20 naturally occurring amino acids were taken from the above mentioned work and were increased by 0.39 if they belonged to an at least 5-residue stretch of alternating polar and non-polar residues. The aggregation propensity of a protein (Pagg) was calculated as:
									(S1)
where piagg is the pagg value of the ith residue along the sequence, and Lprotein the protein length. The aggregation Z-score (Zagg) of a protein has been calculated using:
									(S2)
where agg and agg represent the mean and standard deviation values over a set of 10,000 sequences of length 10,000, generated with amino acid composition deriving from the Swiss-Prot database [2]. We verified that both agg and agg did not depend on protein length (Figure S2), and therefore confidently used constant values of agg and agg for all analysed sequences, equal to 4.2685 and 1.734, respectively. According to this definition, Zagg is a standardized parameter that measures the deviation of the Pagg value from an average value agg, in standard deviation units. Consequently, a Zagg threshold value of 1 constitutes a statistically valid limit to set up the significance of the calculation.

Intrinsic aggregation propensity profile (Zprofagg)
We calculated the aggregation propensity of a 7-residue window centered at position i (Piagg) using:
									(S3)
where pi+jagg is the pagg value of the individual amino acid at position i+j (with j varying from –3 to +3). The Piagg values were not calculated for the first three residues at the N-terminus and the last three residues at the C-terminus, due to the chosen window. We then calculated the intrinsic Z-score of aggregation of a 7-residue window centered at position i (Ziagg) using:
									(S4)
The plot of Ziagg versus residue number (from residue 4 to residue N-3) represents the intrinsic aggregation propensity profile (Zprofagg), as reported [1].

Additional descriptors of the profiles (fpeaks, Lpeaks, Sagg)
A region of a protein sequence was considered aggregation-prone and termed as "peak" whenever Ziagg raised above  for at least three consecutive residues. We introduced parameters to describe the frequency (fpeaks) and the average length (Lpeaks) of the peaks. These two parameters are normalized on protein length using:
									(S5)
									(S6)
where Npeaks is the number of peaks in the sequence, and Li the length of the ith peak of the sequence.
The total surface of the aggregation propensity profile above a threshold of Ziagg = 1 (Sagg) was calculated as previously described [2] with:
									(S7)
where 1 is 1 if ziagg ≥  and 0 if ziagg < . In practice, Sagg measures the area of the profile above  and is highly influenced by both the composition in amino acids and their clustering along the sequence. Figure 1 of the main text reports a scheme of parameters placement on a typical protein profile.

Proteins forming amyloid-like fibrils in vivo in the context of human diseases
>2-microglobulin
IQRTPKIQVYSRHPAENGKSNFLNCYVSGFHPSDIEVDLLKNGERIEKVEHSDLSFSKDWSFYLLYYTEFTPTEKDEYACRVNHVTLSQPKIVKWDRDM 
>A-Dan
EASNCFAIRHFENKFAVETLICFNLFLNSQEKHY
>A-Bri
EASNCFAIRHFENKFAVETLICSRTVKKNIIEEN
>-synuclein
MDVFMKGLSKAKEGVVAAAEKTKQGVAEAAGKTKEGVLYVGSKTKEGVVHGVATVAEKTKEQVTNVGGAVVTGVTAVAQKTVEGAGSIAAATGFVKKDQLGKNEEGAPQEGILEDMPVDPDNEAYEMPSEEGYQDYEPEA
>A1-42
DAEFRHDSGYEVHHQKLVFFAEDVGSNKGAIIGLMVGGVVIA
>IAPP
KCNTATCATQRLANFLVHSSNNFGAILSSTNVGSNTY
>apoAI
DEPPQSPWDRVKDLATVYVDVLKDSGRDYVSQFEGSALGKQLNLKLLDNWDSVTSTFSKLREQLGPVTQEFWDNLEKETEGLRQEMSKDLEEV
>-ANF
SLRRSSCFGGRMDRIGAQSGLGCNSFRY
>calcitonin
CGNLSTCMLGTYTQDFNKFHTFPQTAIGVGAP
>C-crystallin_R168W
GKITFYEDRAFQGRSYETTTDCPNLQPYFSRCNSIRVESGCWMLYERPNYQGQQYLLRRGEYPDYQQWMGLSDSIRSCCLIPQTVSHRLRLYEREDHKGLMMELSEDCPSIQDRFHLSEIRSLHVLEGCWVLYELPNYRGRQYLLRPQEYRRCQDWGAMDAKAGSLRWVVDLY
>cystatin-C
SSPGKPPRLVGGPMDASVEEEGVRRALDFAVGEYNKASNDMYHSRALQVVRARKQIVAGVNYFLDVELGRTTCTKTQPNLDNCPFHDQPHLKRKAFCSFQIYAVPWQGTMTLSKSTCQDA
>Gelsolin
ATEVPVSWESFNNGNCFILDLGNNIHQWCGSNSNRYERLKATQVSKGIRDNERSGRARVHVSEEGTEPEAM 
>Ig_REC
DIVMTQSPDSLAVSLGERATINCKSSQSVLYSSNNRNYLAWYQQKLGQPPKLLIYWASTRESGVPDRFSGSGSGTDFTLISSLQAEDVAVYYCHQYYSHPQTFGQGTKLELKR
>insulin
GIVEQCCTSICSLYQLENYCNFVNQHLCGSHLVEALYLVCGERGFFYTPKT
>Lithostathine
ISCPEGTNAYRSYCYYFNEDRETWVDADLYCQNMNSGNLVSVLTQAEGAFVASLIKESGTDDFNVWIGLHDPKKNRRWHWSSGSLVSYKSWGIGAPSSVNPGYCVSLTSSTGFQKWKDVPCEDKFSFVCKFKN
>Lysozyme
KVFERCELARTLKRLGMDGYRGISLANWMCLAKWESGYNTRATNYNAGDRSTDYGIFQINSRYWCNDGKTPGAVNACHLSCSALLQDNIADAVACAKRVVRDPQGIRAWVAWRNRCQNRDVRQYVQGCGV
>medin
RLDKQGNFNAWVAGSYGNDQWLQVDLGSSKEVTGIITQGARNFGSVQFVA
>PrPc
MLVLFVATWSDLGLCKKRPKPGGWNTGGSRYPGQGSPGGNRYPPQGGGGWGQPHGGGWGQPHGGGWGQPHGGGWGQPHGGGWGQGGGTHSQWNKPSKPKTNMKHMAGAAAAGAVVGGLGGYMLGSAMSRPIIHFGSDYEDRYYRENMHRYPNQVYYRPMDEYSNQNNFVHDCVNITIKQHTVTTTTKGENFTETDVKMMERVVEQMCITQYERESQAYYQRGSSMVLFSSPPVILLISFLIFLIVG
>SAA
RSFFSFLGEAFDGARDMWRAYSDMREANYIGSDKYFHARGNYDAAKRGPGGVWAAEAISDARENIQRFFGHGAEDS 
>SOD-1
ATKAVCVLKGDGPVQGIINFEQKESNGPVKVWGSIKGLTEGLHGFHVHEFGDNTAGCTSAGPHFNPLSRKHGGPKDEERHVGDLGNVTADKDGVADVSIEDSVISLSGDHCIIGRTLVVHEKADDLGKGGNEESTKTGNAGSRLACGVIGIAQ
>Tau
AEPRQEFEVMEDHAGTYGLGDRKDQGGYTMHQDQEGDTDAGLKESPLQTPTEDGSEEPGSETSDAKSTPTAEDVTAPLVDEGAPGKQAAAQPHTEIPEGTTAEEAGIGDTPSLEDEAAGHVTQEPESGKVVQEGFLREPGPPGLSHQLMSGMPGAPLLPEGPREATRQPSGTGPEDTEGGRHAPELLKHQLLGDLHQEGPPLKGAGGKERPGSKEEVDEDRDVDESSPQDSPPSKASPAQDGRPPQTAAREATSIPGFPAEGAIPLPVDFLSKVSTEIPASEPDGPSVGRAKGQDAPLEFTFHVEITPNVQKEQAHSEEHLGRAAFPGAPGEGPEARGPSLGEDTKEADLPEPSEKQPAAAPRGKPVSRVPQLKARMVSKSKDGTGSDDKKAKTSTRSSAKTLKNRPCLSPKLPTPGSSDPLIQPSSPAVCPEPPSSPKHVSSVTSRTGSSGAKEMKLKGADGKTKIATPRGAAPPGQKGQANATRIPAKTPPAPKTPPSSGEPPKSGDRSGYSSPGSPGTPGSRSRTPSLPTPPTREPKKVAVVRTPPKSPSSAKSRLQTAPVPMPDLKNVKSKIGSTENLKHQPGGGKVQIINKKLDLSNVQSKCGSKDNIKHVPGGGSVQIVYKPVDLSKVTSKCGSLGNIHHKPGGGQVEVKSEKLDFKDRVQSKIGSLDNITHVPGGGNKKIETHKLTFRENAKAKTDHGAEIVYKSPVVSGDTSPRHLSNVSSTGSIDMVDSPQLATLADEVSASLAKQGL
>transthyretin
GPTGTGESKCPLMVKVLDAVRGSPAINVAVHVFRKAADDTWEPFASGKTSESGELHGLTTEEEFVEGIYKVEIDTKSYWKALGISPFHEHAEVVFTANDSGPRRYTIAALLSPYSYSTTAVVTNPKE
>lactoferrin
LAGRRRGSVQWCAVSQPEATKCFQWQRNMRRVRGPPVSCIKRDSPIQCIQAIAENRADAVTLDGGFIYEAGLAPYKLRPVAAEVYGTERQPRTHYYAVAVVKKGGSFQLNELQGLKSCHTGLRRTAGWNVPIGTLRPFLNWTGPPEPIEAAVARFFSASCVPGADKGQFPNLCRLCAGTGENKCAFSSQEPYFSYSGAFKCLRDGAGDVAFIRESTVFEDLSDEAERDEYELLCPDNTRKPVDKFKDCHLARVPSHAVVARSVNGKEDAIWNLLRQAQEKFGKDKSPKFQLFGSPSGQKDLLFKDSAIGSSRVPPRIDSGLYLGSGYFTAIQNLRKSEEEVAARRARVVWCAVGEQELRKCNQWSGLSEGSVTCSSASTTEDCIALKGEADAMSLDGGYVYTAGKCGLVPVLAENYKSQQSSDPDPNCVDRPVEGYLAVAVVRRSDTSLTWNSVKGKKSCHTAVDRTAGWNIPMGLLFNQTGSCKFDEYFSQSCAPGSDPRSNLCALCIGDEQGENKCVPNSNERYYGYTGAFRCLAENAGDVAFVKDVTVLQNTDGNNNDAWAKDLKLADFALLCLDGKRKPVTEARSCHLAMAPNHAVVSRMDKVERLKQVLLHQQAKFGRNGSDCPDKFCLFQSETKNLLFNDNTECLARLHGKTTYEKYLGPQYVAGITNLKKCSTSPLLEACEFLRK
>prolactin
LPICPGGAARCQVTLRDLFDRAVVLSHYIHNLSSEMFSEFDKRYTHGRGFITKAINSCHTSSLATPEDKEQAQQMNQKDFLSLIVSILRSWNEPLYHLVTEVRGMQEAPEAILSKAVEIEEQTKRLLEGMELIVSQVHPETKENEIYPVWSGLPSLQMADEESRLSAYYNLLHCLRRDSHKIDNYLKLLKCRIIHNNNC
>Lung_surfactant_prot_C
FGIPCCPVHLKRLLIVVVVVVLIVVVIVGALLMGLHM
>glucagon
HSQGTFTSDYSKYLDSRRAQDFVQWLMNT
>kerato-epithelin
GPAKSPYQLVLQHSRLRGRQHGPNVCAVQKVIGTNRKYFTNCKQWYQRKICGKSTVISYECCPGYEKVPGEKGCPAALPLSNLYETLGVVGSTTTQLYTDRTEKLRPEMEGPGSFTIFAPSNEAWASLPAEVLDSLVSNVNIELLNALRYHMVGRRVLTDELKHGMTLTSMYQNSNIQIHHYPNGIVTVNCARLLKADHHATNGVVHLIDKVISTITNNIQQIIEIEDTFETLRAAVAASGLNTMLEGNGQYTLLAPTNEAFEKIPSETLNRILGDPEALRDLLNNHILKSAMCAEAIVAGLSVETLEGTTLEVGCSGDMLTINGKAIISNKDILATNGVIHYIDELLIPDSAKTLFELAAESDVSTAIDLFRQAGLGNHLSGSERLTLLAPLNSVFKDGTPPIDAHTRNLLRNHIIKDQLASKYLYHGQTLETLGGKKLRVFVYRNSLCIENSCIAAHDKRGRYGTLFTMDRVLTPPMGTVMDVLKGDNRFSMLVAAIQSAGLTETLNREGVYTVFAPTNEDFRALQPRERSRLLGDAKELANILKYHIGDEILVSGGIGALVRLKSLQGDKLEVSLKNNVVSVNKEPVAEPDIMATNGVVHVITNVLQPPANRPQERGDELADSALEIFKQASAFSRASQRSVRLAPVYQKLLERMKH
>cytokeratin5
MSRQSSVSFRSGGSRSFSTASAITPSVSRTSFTSVSRSGGGGGGGFGRVSLAGACGVGGYGSRSLYNLGGSKRISISTRGGSFRNRFGAGAGGGYGFGGGAGSGFGFGGGAGGGFGLGGGAGFGGGFGGPGFPVCPPGGIQEVTVNQSLLTPLNLQIDPSIQRVRTEEREQIKTLNNKFASFIDKVRFLEQQNKVLDTKWTLLQEQGTKTVRQNLEPLFEQYINNLRRQLDSIVGERGRLDSELRNMQDLVEDFKNKYEDEINKRTTAENEFVMLKKDVDAAYMNKVELEAKVDALMDEINFMKMFFDAELSQMQTHVSDTSVVLSMDNNRNLDLDSIIAEVKAQYEEIANRSRTEAESWYQTKYEELQQTAGRHGDDLRNTKHEISEMNRMIQRLRAEIDNVKKQCANLQNAIADAEQRGELALKDARNKLAELEEALQKAKQDMARLLREYQELMNTKLALDVEIATYRKLLEGEECRLSGEGVGPVNISVVTSSVSSGYGSGSGYGGGLGGGLGGGLGGGLAGGSSGSYYSSSSGGVGLGGGLSVGGSGFSASSGRGLGVGFGSGGGSSSSVKFVSTTSSSRKSFKS
>apoAII
QAKEPCVESLVSQYFQTVTDYGKDLMEKVKSPELQAEAKSYFEKSKEQLTPLIKKAGTELVNFLSYFVELGTQPATQGSVQTIVFQFQLASRTPTGQS
>apoAIV
EVSADQVATVMWDYFSQLSNNAKEAVEHLQKSELTQQLNALFQDKLGEVNTYAGDLQKKLVPFATELHERLAKD
>Fibrinogen
FDTASTGKTFPGFFSPMLGEFVSETESRGSESGIFTNTKESSSHHPGIAEFPSRGKSSSYSKQFTSSTSYNRGDSTFESK

Proteins forming amyloid-like firbils in vivo in the context of human diseases, folded
>2-microglobulin
IQRTPKIQVYSRHPAENGKSNFLNCYVSGFHPSDIEVDLLKNGERIEKVEHSDLSFSKDWSFYLLYYTEFTPTEKDEYACRVNHVTLSQPKIVKWDRDM 
>C-crystallin_R168W
GKITFYEDRAFQGRSYETTTDCPNLQPYFSRCNSIRVESGCWMLYERPNYQGQQYLLRRGEYPDYQQWMGLSDSIRSCCLIPQTVSHRLRLYEREDHKGLMMELSEDCPSIQDRFHLSEIRSLHVLEGCWVLYELPNYRGRQYLLRPQEYRRCQDWGAMDAKAGSLRWVVDLY
>cystatin-C
SSPGKPPRLVGGPMDASVEEEGVRRALDFAVGEYNKASNDMYHSRALQVVRARKQIVAGVNYFLDVELGRTTCTKTQPNLDNCPFHDQPHLKRKAFCSFQIYAVPWQGTMTLSKSTCQDA
>Ig_REC
DIVMTQSPDSLAVSLGERATINCKSSQSVLYSSNNRNYLAWYQQKLGQPPKLLIYWASTRESGVPDRFSGSGSGTDFTLISSLQAEDVAVYYCHQYYSHPQTFGQGTKLELKR
>insulin
GIVEQCCTSICSLYQLENYCNFVNQHLCGSHLVEALYLVCGERGFFYTPKT
>Lithostathine
ISCPEGTNAYRSYCYYFNEDRETWVDADLYCQNMNSGNLVSVLTQAEGAFVASLIKESGTDDFNVWIGLHDPKKNRRWHWSSGSLVSYKSWGIGAPSSVNPGYCVSLTSSTGFQKWKDVPCEDKFSFVCKFKN
>Lysozyme
KVFERCELARTLKRLGMDGYRGISLANWMCLAKWESGYNTRATNYNAGDRSTDYGIFQINSRYWCNDGKTPGAVNACHLSCSALLQDNIADAVACAKRVVRDPQGIRAWVAWRNRCQNRDVRQYVQGCGV
>medin
RLDKQGNFNAWVAGSYGNDQWLQVDLGSSKEVTGIITQGARNFGSVQFVA
>SAA
RSFFSFLGEAFDGARDMWRAYSDMREANYIGSDKYFHARGNYDAAKRGPGGVWAAEAISDARENIQRFFGHGAEDS 
>SOD-1
ATKAVCVLKGDGPVQGIINFEQKESNGPVKVWGSIKGLTEGLHGFHVHEFGDNTAGCTSAGPHFNPLSRKHGGPKDEERHVGDLGNVTADKDGVADVSIEDSVISLSGDHCIIGRTLVVHEKADDLGKGGNEESTKTGNAGSRLACGVIGIAQ
>transthyretin
GPTGTGESKCPLMVKVLDAVRGSPAINVAVHVFRKAADDTWEPFASGKTSESGELHGLTTEEEFVEGIYKVEIDTKSYWKALGISPFHEHAEVVFTANDSGPRRYTIAALLSPYSYSTTAVVTNPKE
>lactoferrin
LAGRRRGSVQWCAVSQPEATKCFQWQRNMRRVRGPPVSCIKRDSPIQCIQAIAENRADAVTLDGGFIYEAGLAPYKLRPVAAEVYGTERQPRTHYYAVAVVKKGGSFQLNELQGLKSCHTGLRRTAGWNVPIGTLRPFLNWTGPPEPIEAAVARFFSASCVPGADKGQFPNLCRLCAGTGENKCAFSSQEPYFSYSGAFKCLRDGAGDVAFIRESTVFEDLSDEAERDEYELLCPDNTRKPVDKFKDCHLARVPSHAVVARSVNGKEDAIWNLLRQAQEKFGKDKSPKFQLFGSPSGQKDLLFKDSAIGSSRVPPRIDSGLYLGSGYFTAIQNLRKSEEEVAARRARVVWCAVGEQELRKCNQWSGLSEGSVTCSSASTTEDCIALKGEADAMSLDGGYVYTAGKCGLVPVLAENYKSQQSSDPDPNCVDRPVEGYLAVAVVRRSDTSLTWNSVKGKKSCHTAVDRTAGWNIPMGLLFNQTGSCKFDEYFSQSCAPGSDPRSNLCALCIGDEQGENKCVPNSNERYYGYTGAFRCLAENAGDVAFVKDVTVLQNTDGNNNDAWAKDLKLADFALLCLDGKRKPVTEARSCHLAMAPNHAVVSRMDKVERLKQVLLHQQAKFGRNGSDCPDKFCLFQSETKNLLFNDNTECLARLHGKTTYEKYLGPQYVAGITNLKKCSTSPLLEACEFLRK
>prolactin
LPICPGGAARCQVTLRDLFDRAVVLSHYIHNLSSEMFSEFDKRYTHGRGFITKAINSCHTSSLATPEDKEQAQQMNQKDFLSLIVSILRSWNEPLYHLVTEVRGMQEAPEAILSKAVEIEEQTKRLLEGMELIVSQVHPETKENEIYPVWSGLPSLQMADEESRLSAYYNLLHCLRRDSHKIDNYLKLLKCRIIHNNNC
>kerato-epithelin 
GPAKSPYQLVLQHSRLRGRQHGPNVCAVQKVIGTNRKYFTNCKQWYQRKICGKSTVISYECCPGYEKVPGEKGCPAALPLSNLYETLGVVGSTTTQLYTDRTEKLRPEMEGPGSFTIFAPSNEAWASLPAEVLDSLVSNVNIELLNALRYHMVGRRVLTDELKHGMTLTSMYQNSNIQIHHYPNGIVTVNCARLLKADHHATNGVVHLIDKVISTITNNIQQIIEIEDTFETLRAAVAASGLNTMLEGNGQYTLLAPTNEAFEKIPSETLNRILGDPEALRDLLNNHILKSAMCAEAIVAGLSVETLEGTTLEVGCSGDMLTINGKAIISNKDILATNGVIHYIDELLIPDSAKTLFELAAESDVSTAIDLFRQAGLGNHLSGSERLTLLAPLNSVFKDGTPPIDAHTRNLLRNHIIKDQLASKYLYHGQTLETLGGKKLRVFVYRNSLCIENSCIAAHDKRGRYGTLFTMDRVLTPPMGTVMDVLKGDNRFSMLVAAIQSAGLTETLNREGVYTVFAPTNEDFRALQPRERSRLLGDAKELANILKYHIGDEILVSGGIGALVRLKSLQGDKLEVSLKNNVVSVNKEPVAEPDIMATNGVVHVITNVLQPPANRPQERGDELADSALEIFKQASAFSRASQRSVRLAPVYQKLLERMKH
>cytokeratin5
MSRQSSVSFRSGGSRSFSTASAITPSVSRTSFTSVSRSGGGGGGGFGRVSLAGACGVGGYGSRSLYNLGGSKRISISTRGGSFRNRFGAGAGGGYGFGGGAGSGFGFGGGAGGGFGLGGGAGFGGGFGGPGFPVCPPGGIQEVTVNQSLLTPLNLQIDPSIQRVRTEEREQIKTLNNKFASFIDKVRFLEQQNKVLDTKWTLLQEQGTKTVRQNLEPLFEQYINNLRRQLDSIVGERGRLDSELRNMQDLVEDFKNKYEDEINKRTTAENEFVMLKKDVDAAYMNKVELEAKVDALMDEINFMKMFFDAELSQMQTHVSDTSVVLSMDNNRNLDLDSIIAEVKAQYEEIANRSRTEAESWYQTKYEELQQTAGRHGDDLRNTKHEISEMNRMIQRLRAEIDNVKKQCANLQNAIADAEQRGELALKDARNKLAELEEALQKAKQDMARLLREYQELMNTKLALDVEIATYRKLLEGEECRLSGEGVGPVNISVVTSSVSSGYGSGSGYGGGLGGGLGGGLGGGLAGGSSGSYYSSSSGGVGLGGGLSVGGSGFSASSGRGLGVGFGSGGGSSSSVKFVSTTSSSRKSFKS

Proteins forming amyloid-like fibrils in vitro although not involved in diseases
>mAcP
STAQSLKSVDYEVFGRVQGVCFRMYTEDEARKIGVVGWVKNTSKGTVTGQVQGPEDKVNSMKSWLSKVGSPSSRIDRTNFSNEKTISKLEYSNFSIRY
>apoCII
MGTRLLPALFLVLLVLGFEVQGTQQPQQDEMPSPTFLTQVKESLSSYWESAKTAAQNLYEKTYLPAVDEKLRDLYSKSTAAMSTYTGIFTDQVLSVLKGEE
>endostatin
HTHQDFQPVLHLVALNTPLS
>27thIg_domain_Titin
PSFVKKVDPSYLMLPGESARLHCKLKGSPVIQVTWFKNNKELSESNTVRMYFVNSEAILDITDVKVEDSGSYSCEAVNDVGSDSCSTEIV
>exon30_elastin
GLVGAAGLGGLGVGGLGVPGVGGLG
>ADA2h
MRSLETFVGDQVLEIVPSNEEQIKNLLQLEAQEHLQLDFWKSPTTPGETAHVRVPFVNVQAVKVFLESQGIAYSIMIEDVQ
>histone_H4
SGRGKGGKGLGKGGAKRHRKVLRDNIQGITKPAIRRLARRGGVKRISGLIYEETRGVLKVFLENVIRDAVTYTEHAKRKTVTAMDVVYALKRQGRTLYGFGG
>2ndWW_CA150
ATAVSEWTEYKTADGKTYYYNNRTLESTWEKPQE
>Cystatin_B
MMCGAPSATQPATAETQHIADQVRSQLEEKENKKFPVFKAVSFKSQVVAGTNYFIKVHVGDEDFVHLRVFQSLPHENKPLTLSNYQTNKAKHDELTYF
>pancreatitis-associated protein
EEPQRELPSARIRCPKGSKAYGSHCYALFLSPKSWTDADLACQKRPSGNLVSVLSGAEGSFVSSLVKSIGNSYSYVWIGLHDPTQGTEPNGEGWEWSSSDVMNYFAWERNPSTISSPGHCASLSRSTAFLRWKDYNCNVRLPYVCKFTD


References for Protocol S1

1. Pawar AP, DuBay KF, Zurdo J, Chiti F, Vendruscolo M, et al. (2005) Prediction of "aggregation-prone" and "aggregation-susceptible" regions in proteins associated with neurodegenerative diseases. J Mol Biol 350: 379-392.
2. Monsellier E, Ramazzotti M, Polverino de Laureto P, Tartaglia GG, Taddei N, et al. (2007) The distribution of residues in a polypeptide sequence is a determinant of aggregation optimized by evolution. Biophys J 93: 4382-4391.
